# The Gut Microbiota and Developmental Programming of the Testis in Mice

**DOI:** 10.1371/journal.pone.0103809

**Published:** 2014-08-13

**Authors:** Maha Al-Asmakh, Jan-Bernd Stukenborg, Ahmed Reda, Farhana Anuar, Mona-Lisa Strand, Lars Hedin, Sven Pettersson, Olle Söder

**Affiliations:** 1 Department of Microbiology, Tumor and Cell Biology, Karolinska Institutet, Stockholm, Sweden; 2 Department of Biomedical Science, College of Arts and Sciences, Qatar University, Doha, Qatar; 3 Department of Women's and Children's Health, Paediatric Endocrinology Unit, Karolinska Institutet and University Hospital, Stockholm, Sweden; 4 LKC School of Medicine, Nanyang Technological University, Singapore, Singapore; 5 Sidra Medical and Research Center, Division of Clinical Epidemiology, Doha, Qatar; University of Cape Town, South Africa

## Abstract

Nutrients and environmental chemicals, including endocrine disruptors, have been incriminated in the current increase in male reproductive dysfunction, but the underlying mechanisms remain unknown. The gastrointestinal tract represents the largest surface area exposed to our environment and thereby plays a key role in connection with exposure of internal organs to exogenous factors. In this context the gut microbiome (all bacteria and their metabolites) have been shown to be important contributors to body physiology including metabolism, cognitive functions and immunity. Pivotal to male reproduction is a proper development of the testis, including the formation of the blood-testis barrier (BTB) that encapsulates and protects germ cells from stress induced environmental cues, e.g. pathogenic organisms and xenobiotics. Here we used specific pathogen free (SPF) mice and germ-free (GF) mice to explore whether gut microbiota and/or their metabolites can influence testis development and regulation of BTB. Lumen formation in the seminiferous tubules, which coincides with the development of the BTB was delayed in the testes of GF mice at 16 days postpartum. In addition, perfusion experiments (Evans blue) demonstrated increased BTB permeability in these same mice. Reduced expressions of occludin, ZO-2 and E-cadherin in GF testis suggested that the microbiota modulated BTB permeability by regulation of cell-cell adhesion. Interestingly, exposure of GF mice to *Clostridium Tyrobutyricum* (CBUT), which secrete high levels of butyrate, restored the integrity of the BTB and normalized the levels of cell adhesion proteins. Moreover, the GF mice exhibited lower serum levels of gonadotropins (LH and FSH) than the SPF group. In addition, the intratesticular content of testosterone was lower in GF compared to SPF or CBUT animals. Thus, the gut microbiome can modulate the permeability of the BTB and might play a role in the regulation of endocrine functions of the testis.

## Introduction

The blood-testis barrier (BTB), considered to be one of the tightest epithelial barriers in the entire human body, is formed by specialized junctions between adjacent Sertoli cells located near the basement membrane of the seminiferous epithelium. Unlike the interstitial compartment, it is devoid of capillaries, nerves and lymphatic vessels [1]. During spermatogenesis, the BTB undergoes cyclic restructuring with preleptotene spermatocytes migrating into the adluminal compartment and entering meiosis [2], [3]. Thus, the BTB divides the seminiferous epithelium into a distinct basal compartment, which provides the niche for the proliferation and renewal of spermatogonia, and a separate adluminal compartment, where meiosis and spermiogenesis occur [2]. Accordingly, the BTB constitutes an immunological and physical barrier that segregates postmeiotic germ cells from the host immune system and hinders the entry of leukocytes and antibodies into the adluminal compartment of the testis [4]. The tight junction proteins (TJPs) that bridge Sertoli cells are composed of occludin, claudins and junctional adhesion molecules (JAM), which are linked to the cytoskeleton by zonula occludens proteins (ZO-1, ZO-2 and ZO-3) [5] and effectively restrict paracellular passage of molecules [6], [7].

The increasing incidence of testicular abnormalities has been proposed to involve exogenous factors [8], [9] with resulting impaired fertility. The gastrointestinal tract not only mediates efficient uptake of nutrients, but also of exogenous factors, including invasive microorganisms with potentially harmful actions on the testis. The local effects of the gut microbiota on intestinal development and function are well documented. The gut microbiota is involved in the establishment of the physical barrier of the gut epithelium, in the local regulation of the immune system and also in the modulation of metabolic processes [10], [11]. We have previously shown that the gut microbiota can exert systemic effects on organs outside the gastrointestinal tract, e.g. the brain with effects on behavior [12] and on the permeability of the blood-brain barrier (Braniste *et al.*, in revised version) [13].

Diet and environmental factors have been shown to affect testicular steroidogenesis [14], [15]. The synthesis of testosterone by Leydig cells requires the action of several enzymes which fall into two categories: the cytochrome P450 enzymes and the hydroxysteroid dehydrogenases (HSD) [16]. Earlier studies have documented that the gut microbiota is involved in the deconjugation and metabolism of steroid hormones [17]. In the present study we demonstrate that the gut microbiota affect the testis- pituitary axis, regulates lumen formation of the seminiferous tubules and the cell-cell adhesion and also the permeability of the BTB.

## Materials and Methods

### Animals and Treatments

Male NMRI mice were maintained on a standard R36 chow (Lactamin, Sweden) with a 12-h light cycle. These animals were raised either under germ-free (GF) conditions in stainless steel isolators, or under conventional conditions in a specific pathogen-free environment (SPF). Mice from at least three different litters were sacrificed either 16 days *post partum* (d*pp*) or as adults (8–10 weeks *post partum*) and their testicular tissues were either fixed immediately in 4% paraformaldehyde (PFA; 8187081000, Merck) or snap-frozen in liquid nitrogen for further analysis as described below. All experiments were performed using male NMRI mice. The permeability test (Evans blue) was done on C57Bl/6J and NMRI mice, in order to confirm that the increase in permeability in GF mice was not strain dependent.

#### Colonization of GF mice with bacteria

GF mice received (1×10^8^ bacteria per mouse) of *Clostridium Tyrobutyricum* (CBUT) suspended in autoclaved phosphate buffered saline (PBS) by oral gavage at least 30 days prior to mating and their pups were subsequently sacrificed at 16d*pp*. Nine animals (from three different litters) were used in each experimental group (adult and 16d*pp*; SPF, GF and CBUT). CBUT bacteria was a kind gift from Dr. Hana Kozakova, Department of Immunology and Gnotobiology – Institute of Microbiology – Praha, The Czech Republic [18].

Adult mice were anesthetized with isoflurane and sacrificed by cervical dislocation, while 16d*pp* pups were sacrificed by decapitation. All experiments were pre-approved by the local Committee on the Ethics of Animal Experimentation, Stockholm, Sweden (N530-12006).

### Sperm Counts

Caudal epididymides were minced in 1 ml PBS and the total sperm count determined using a hemocytometer as described previously [19].

### Immunohistochemistry and staining with Periodic acid-Schiff (PAS)

The testes were fixed immediately in 4% PFA (8187081000, Merck, Germany) at 4°C overnight, then serially dehydrated with 30%, 50%, and 70% ethanol for 24 h and embedded in paraffin using standard procedures. Five µm sections were either used for immunohistochemistry or stained with PAS (1.01644, Merck, Germany) in accordance with the manufacturer's protocol. For immunohistochemistry staining, the paraffin embedded samples were de-paraffinized using Xylene (02080, Histolab, Sweden) for 10 min and rehydrated in descending ethanol gradient (100 – 96 – 70%) solutions. Meanwhile, blocking of endogenous peroxidase was done for 10 min during rehydration in 96% ethanol containing 0.3% H_2_O_2_ (1.07209.250, Merck, Germany). Antigen retrieval was performed by addition of sodium citrate buffer, pH 6.0 (C7254, Sigma–Aldrich, U.S.A.) at 95°C for 30 min and slides were allowed to cool for another 30 min. Blocking with 10% goat serum (S-1000, Vector laboratories, U.S.A) and 1% bovine serum albumin (BSA; 001.000-0162, Jackson Immunoresearch, U.S.A) in PBS was performed for 1 h at room temperature. Sections were then incubated with a monoclonal rabbit anti-vimentin primary antibody (ab92547, Abcam, UK, 1∶200 dilution) or rabbit IgGs for negative control (ab27478, Abcam, UK, 1∶50 dilution), in a PBS buffer containing 10% goat serum and 1% BSA overnight at 4°C. After washing with PBS, sections were incubated with horseradish-peroxidase conjugated goat anti-rabbit secondary antibody (ab6721, Abcam, UK, 1∶400 dilutions) for 1 h at room temperature. This was followed by rinsing in PBS and incubation with diaminobenzidine (DAB; SK-4105, Vector Laboratories, U.S.A) with subsequent development after 1 min according to the manufacturer's protocol. The slides were counterstained with haematoxyline (1.09249.1000, Merck, Germany), dehydrated in ascending ethanol (70 – 96 – 100%) and xylene, and mounted in Petrex non-aqueous medium (00871.0500, Histolab, Sweden). 55 to 57 seminiferous tubules from 5–6 animals/per group (SPF, GF or CBUT) were analyzed. The sections were analyzed with an Eclipse E800 microscope (Nikon, Japan) with photographic documentation using a cooled 12.5 million-pixel digital color camera system (Olympus DP70, Japan). Counting was performed manually.

For immunofluorescence staining, the slides were thawed at room temperature before rehydration with PBS for 5 min, then fixed with ice-cold acetone for 10 min, blocked and permeabilized for 45 min with PBS containing 1.5% BSA, 5% donkey serum and 0.05% Triton X-100 (Sigma–Aldrich, U.S.A.). After three subsequent washes with PBS, these tissue sections were incubated with the primary antibody overnight at 4°C, washed again; and then incubated for 1 h at room temperature in the dark with the secondary antibody. Finally, the slides were washed, mounted with Vectra shield containing DAPI (H-1200, Vector Laboratories, U.S.A.) and stored at 4°C in the dark. Controls for specificity and background staining were performed as described above, but the specific primary antibodies were replaced with mouse IgG or rabbit IgG antibodies.

### Determination of the tubular diameter and luminal opening

Sections (5 µm) of paraffin-embedded testes stained with PAS, were used to determine the diameter of the seminiferous tubules (average of five separate measurements on each lumen) and the proportion of opened lumens (n = 9; 16d*pp*, or n = 5–6; adult) in each of the groups from three different litters, were measured (blinded) using a light microscopy (magnification 40×).

### Evaluation of the physical integrity of the BTB by perfusion with Evans blue

The thoracic cavity of isoflurane-anesthetized mice was opened and a 25 gauge needle for perfusion was inserted into the left ventricle. Thereafter, incoming venous blood was drained through an incision in the right auricle and the perfusion solution allowed to enter the left ventricle at a mean rate of approximately 7 ml/min. Perfusion with 50 ml of PBS (pH 7.2) was followed by 50 ml of the Evans blue cocktail (1% Evans blue and 4% PFA) in PBS. Thus, each mouse received 500 mg of Evans blue (EB), which binds to serum albumin (m_r_ = 65 kDa) [20]. The dissected testes were post-fixed in 4% PFA in PBS overnight at 4°C, followed by immersion in PBS containing 30% sucrose overnight at 4°C for cryoprotection, embedded in OCT cryostat-embedding compound (Tissue-Tek, Torrance, CA, U.S.A) and finally chilled on dry ice and stored at −80°C until further use.

### RNA extraction and RT-qPCR

RNA was isolated with the Qiagen RNeasy Mini-kit (Qiagen, Hilden, Germany) in accordance with the manufacturer's instructions. Briefly, cDNA was synthesized with Super-Script II (Invitrogen, Carlsbad, CA, U.S.A). Oligo-dT primers were used in the presence of RNaseOUT reagent (Invitrogen, Carlsbad, CA, U.S.A), with 2 µg of RNA per reaction. 1 µl cDNA (diluted 1∶5) was subjected to qPCR in the Abi Prism 7500 (Applied Biosystems, Foster City, CA, U.S.A) thermal cycler utilizing the SYBR-Green reagent (Applied Biosystems, Foster City, CA, U.S.A) and gene specific-primers listed in Table S1. The levels of the mRNA species of interest were normalized to the housekeeping levels of *18s rRNA* and *Arbpa/Rplp0* mRNA and analyzed by the 2^−ΔΔCT^ procedure described previously [21]. Six animals were analyzed in each experimental group and the samples were run in duplicates.

### Protein extraction, western blots and protein quantification

Testes were homogenized in ice-cold RIPA lysis buffer in the presence of freshly prepared protease inhibitors. After centrifugation (14,000 rpm, 1 min), aliquots of the supernatant (50 µg of total protein) were mixed with reducing sample buffer and heated to 95°C for 5 min. The denatured samples were then electrophoresed on 7.5–12% SDS-PAGE gels at a constant voltage (130V) and electroblotted onto PVDF membranes (BioRad, Hercules, CA, U.S.A.), and pre-soaked in ice-cold methanol for 1 min. The blots were then incubated in blocking solution (0.1% Tween-20, 5% milk in PBS) for 1 h at room temperature.

Subsequently, primary antibodies (prepared in blocking reagent at the appropriate dilution) were incubated with the blot overnight at 4°C, following which non-specific binding was eliminated by washing (3×10 min) in wash buffer (0.1% Tween in PBS). Next, incubation with horseradish-peroxidase conjugated secondary antibodies (Dako, Glostrup, Denmark), diluted 1∶2000 in the blocking reagent was performed at room temperature for 1 h. After final washes (3×10 min; 0.1% Tween in PBS) the protein bands were visualized by chemiluminescence using Immun-Star WesternC Chemiluminescent Kit (BioRad, Hercules, CA, U.S.A.) and quantified utilizing Bio-Rad Quantity One 1-D analysis software. For a complete list of antibodies, see Table S2.

### Hormone assays

Intra-testicular levels of testosterone were quantified employing the Coat-a-Count RIA Kit (TFTF2, Siemens, Munich, Germany) in accordance with the manufacturer's instructions with intra and inter-assay coefficients of variations of 7.4 and 3.2%, respectively. In brief, testes were weighted, homogenized and sonicated individually (Vibra Cell sonicator [Sonics and Materials Inc., U.S.A], 10 sec, 30% amplitude) in an Eppendorf tube containing 200 µl PBS. Testosterone was then extracted by adding 0.5 ml ethyl acetate (300612, Merck, Germany) to the homogenate, followed by vigorous, automated shaking at 900 rpm for 15 min. Thereafter, the samples were centrifuged for 2 min at 13,000 rpm and the supernatant transferred to a new 1.5 ml Eppendorf tube and left overnight under the hood at room temperature to allow for evaporation of the ethyl acetate.

The resulting pellets, containing testosterone, were dissolved in 100 µl PBS and subjected immediately (in duplicates) to radioimmunoassay (RIA). The samples were aliquoted into tubes containing I^125^ testosterone; then incubated at 37°C for 3 h (shaking at 120 rpm); decanted and followed by measurement of radioactivity for 1 min with a Gamma counter (1470 Wizard Wallac, GMI, Massachusetts, U.S.A.). The total amount of testosterone was calculated in pmoles and expressed as per gram of testis.

LH and FSH in serum were measured by enzyme-linked immunosorbent assay (ELISA) kits (E90441Mu, E90830Mu respectively, Uscn Life Science Inc., Wuhan, China) in accordance with the manufacturer's protocols. In brief, serum samples, together with the standards, were incubated in 96-well plates at 37°C for the time and with the reagents specified for each protocol. The plates were rinsed with washing buffer and incubated again at 37°C, followed by the addition of substrate solution and subsequently stopping buffer. Absorption at 450 nm was measured with the plate reader (Perkin Elmer, Massachusetts, U.S.A.) and serum concentrations of LH and FSH were calculated from the standard curves.

### Statistical analysis

One-way ANOVA analysis with Tukey's post-hoc test (GraphPad Prism 6, San Diego, CA, U.S.A.) was used for statistical analysis of at least three groups. The Mann-Whitney Rank Sum Test and Student's t-test were used to compare two groups. *P*<0.05 was considered to be statistically significant unless otherwise indicated.

## Results

### The gut microbiota exerts no effect on male fertility

**Figure 1:**
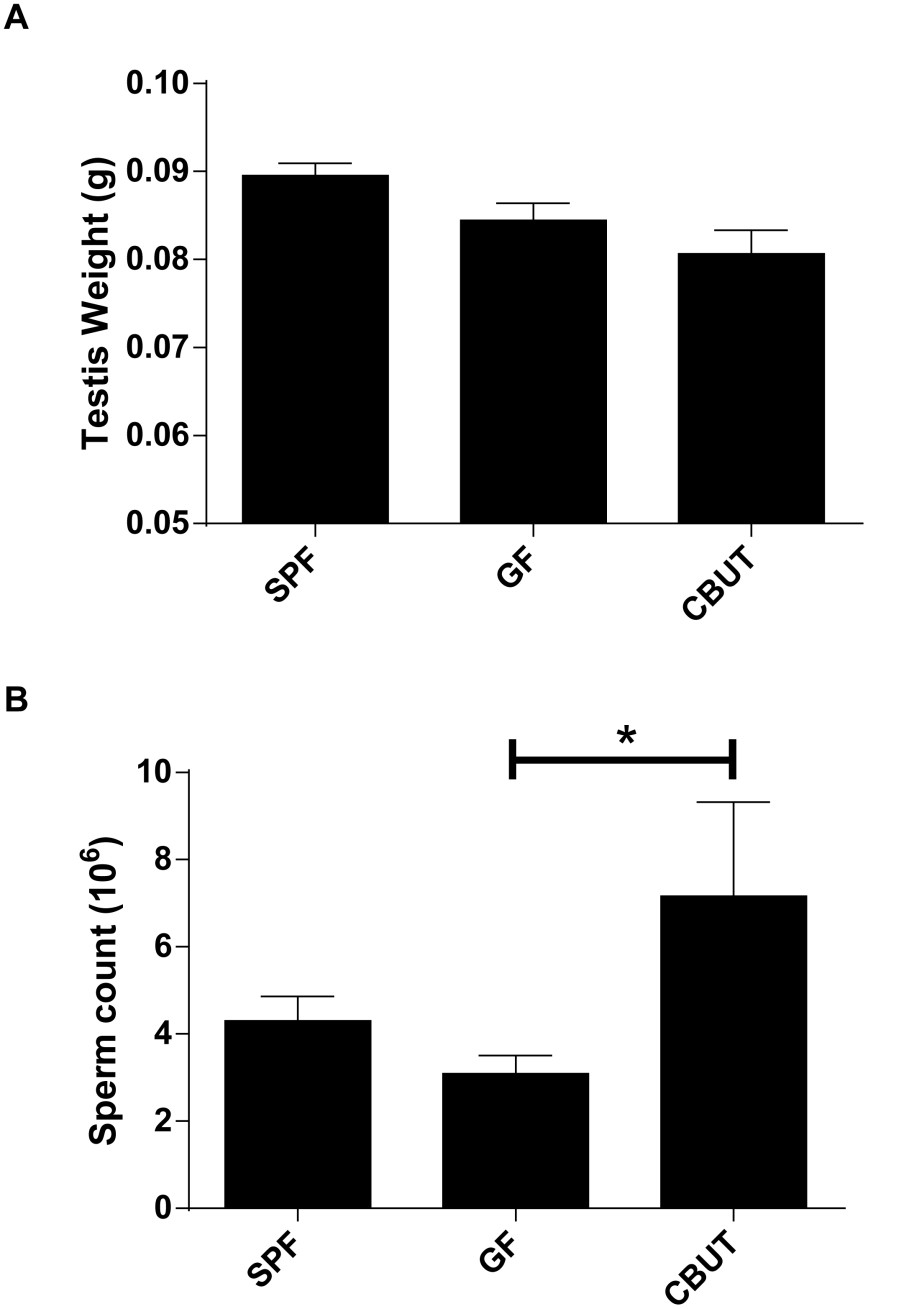
Testis weight (A) and sperm count (B) for SPF, GF and CBUT mice at 8 weeks of age. The values shown are means ± SEM (n = 6) *P<0.05 calculated by the Mann-Whitney Rank Sum Test for non-parametric independent data.

The control group (SPF) and GF mice exhibited similar testis weights and sperm counts (Figure 1A–B) and they were both fertile. The mean litter size did not differ between the groups (SPF = 6.8, GF = 8.07 and CBUT = 7.17). In addition, the sperm counts for CBUT, but not SPF mice were higher than for GF mice (Figures 1B).

### The gut microbiota influences lumen formation in the prepubertal testis of mice

**Figure 2:**
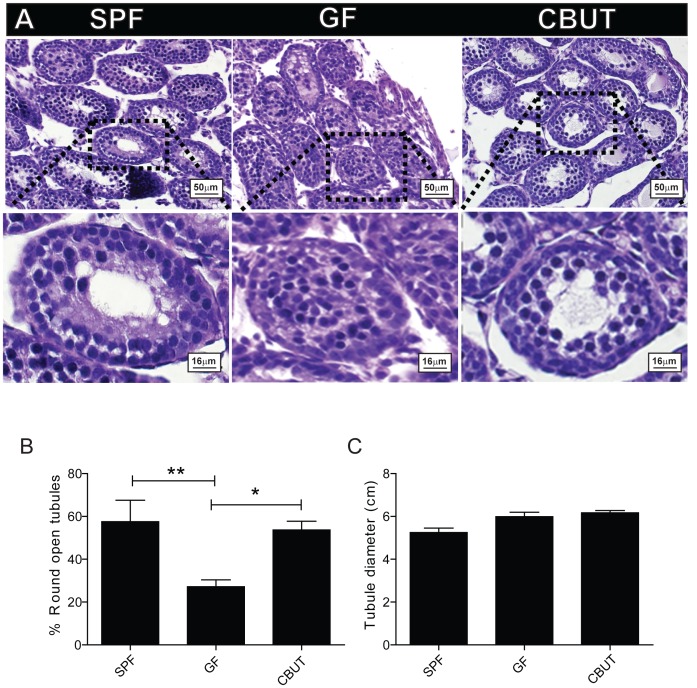
Lumen formation on postnatal day 16. (**A**) Representative PAS staining of seminiferous tubules from the SPF, GF and CBUT mice. (**B**) The percentage of open tubules and (**C**) tubule diameter in the seminiferous tubules of SPF, GF and CBUT mice. The values presented are means ± SEM for 9 mice from 3 different litters *P<0.05 calculated by One-way ANOVA.

**Figure 3:**
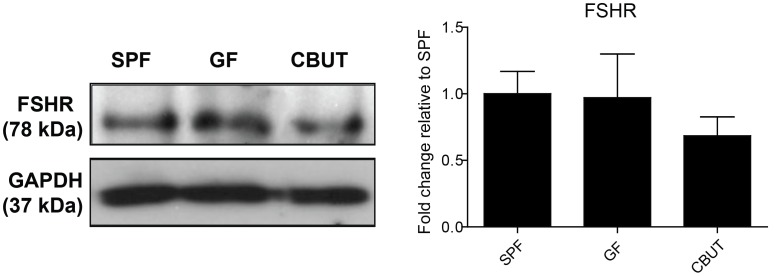
Expression of Sertoli cell markers in the SPF, GF and CBUT testes. Representative Western blots for the Follicle-stimulating hormone receptor (FSHR) and quantification of these blots relative to the levels of Glyceraldehyde 3-phosphate dehydrogenase (GAPDH) (means ± SEM, n = 3–6 *P<0.05) compared to the values for SPF mice.

The BTB is formed by postnatal day 15–16 in mice [22] which coincides with the period when the testis cords are transformed into seminiferous tubules with lumens [23]. PAS staining revealed a higher percentage of still closed seminiferous tubules in the testis of GF- compared to SPF- or CBUT mice, with no difference in tubule diameter (Figure 2). This delayed lumen formation in the GF murine testis can be the result of a reduction in the number of Sertoli cells that forms the BTB. However, there was no difference in the number of Sertoli cells between the adult SPF and GF mice, as assessed by the expression of two specific markers for Sertoli cells: either by quantitative immunoblotting of the follicle-stimulating hormone receptor (FSHR; Figure 3), or immunohistochemical analysis of cells staining positive for vimentin (Figure S1) (7.23%±1.18, 7.12%±1.03, respectively). However, there was significantly higher percentage of Sertoli cells per tubule positive for vimentin in the CBUT group (9.09%±3.12) compared to both the GF- and SPF groups (Figure S1). Nevertheless, this difference was not observed for the expression of FSHR which was similar in all three experimental groups (Figure 3).

### The gut microbiota is involved in the regulation of cell-cell adhesion of Sertoli cells in the BTB

**Figure 4:**
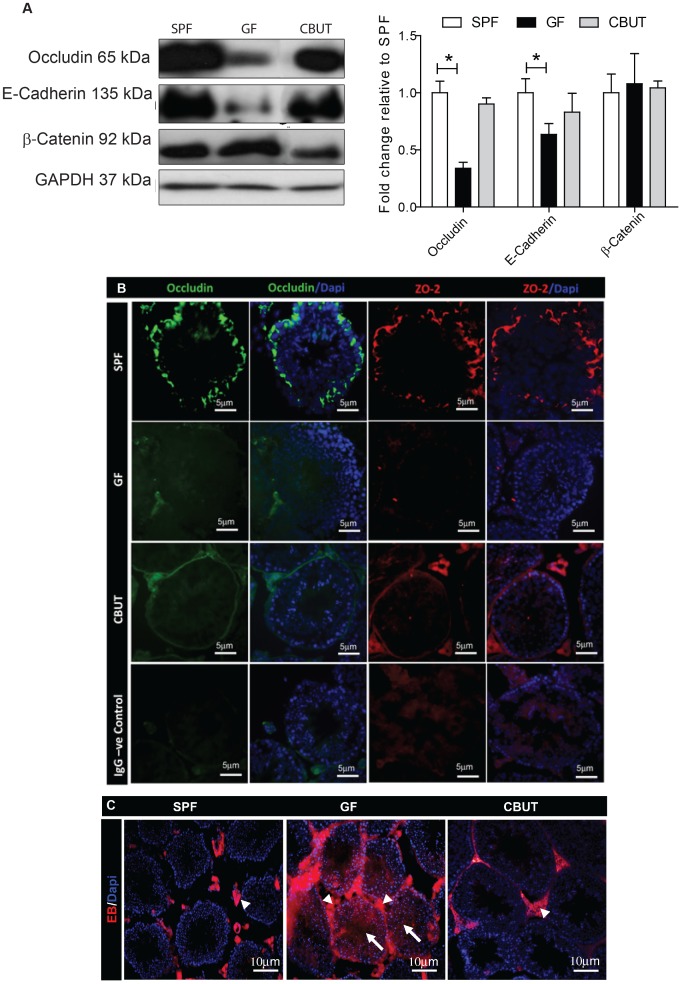
Expression of tight junction proteins and changes in the permeability of the BTB of 8–10 weeks old SPF, GF and CBUT mice. (**A**) Representative Western blots for occludin, E-cadherin and β-catenin, and quantification of these blots relative to the levels of GAPDH (means ± SEM., n = 3–6 *P<0.05) compared to the values for SPF mice. (**B**) Immunofluorescent staining for occludin (green) and ZO-2 (red) individually and merged with nuclear staining (DAPI – blue). The scale bar represents 5 µm. (**C**) Evans blue (EB – red) and nuclear staining (DAPI – blue) showing interstitial cells (arrowheads) and seminiferous tubules (arrows). Scale bar represents 10 µm.

The paracellular permeability of the BTB is regulated by cell-cell adhesion between Sertoli cells, formed mainly by adherens junctions (AJ) and tight junctions (TJ). The expressions of occludin (Figure 4A and B), ZO-2 (Figure 4B) (both components of TJ) and E-cadherin (component of AJ) (Figure 4A) were all lower in the testis of GF compared to SPF mice. Colonization with CBUT restored the expression levels of occludin (Figures 4A and B), ZO-2 (Figure 4B) and E-cadherin (Figure 4A). In contrast, β-catenin (an adaptor protein of AJ), which is involved in intracellular signaling of the AJ was not affected by the gut microbiota (Figure 4A).

### The gut microbiota affect the permeability of the BTB

Since the previous experiments had demonstrated reduction of components of AJ and TJ, the physical properties of the blood-testis barrier (BTB) in GF and SPF mice were assessed by perfusion with Evans blue (EB). Positive fluorescence (bright red) between Sertoli cells and in the lumen of seminiferous tubules was observed in GF mice (Figure 4C). In contrast, in the SPF and CBUT mice, fluorescence was detected only in the interstitium, and not in the lumen of the seminiferous tubules, demonstrating the presence of a functional, non-permeable BTB (Figure 4C).

### The gut microbiota influence the intra-testicular levels of testosterone and serum levels of gonadotropins

**Figure 5:**
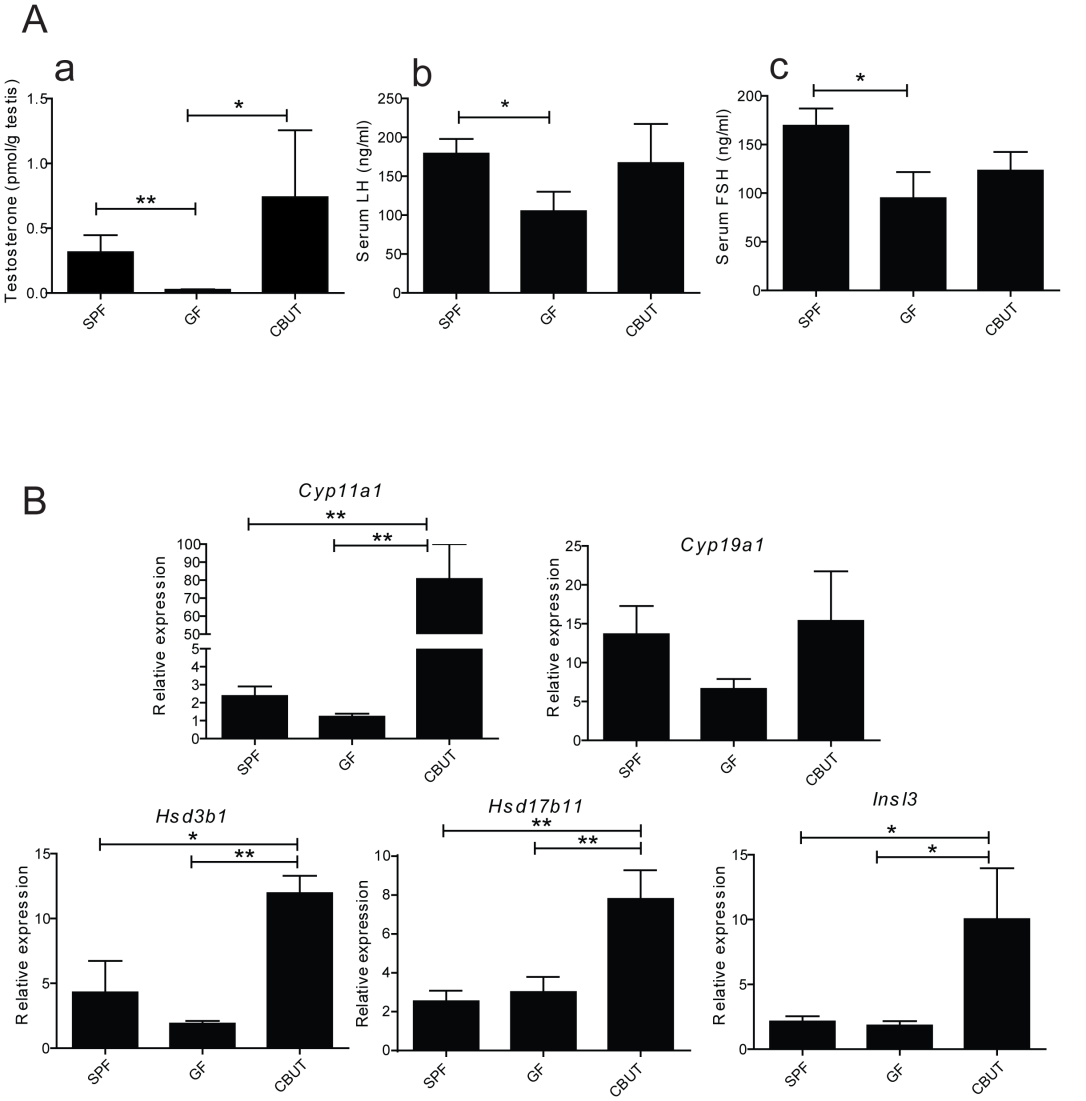
Assessment of hormone levels and mRNA levels encoding steroidogenic proteins. (**A**) Intratesticular levels of testosterone and serum levels of LH and FSH in 8–10 weeks old SFP, GF and CBUT mice. (a) Levels of intra-testicular testosterone per gram testis weight measured with RIA. Each bar represents the mean ± SEM (n = 6–9). The actual values were 0.315±0.131 pmol/g for SPF mice, 0.025±0.003 pmol/g for the GF group and 0.739±0.517 pmol/g for CBUT mice *P<0.05, **<0.001 as determined by the Mann-Whitney Rank Sum Test for non-parametric independent data. (b) Levels of serum LH as determined by ELISA. Each bar represents the mean ± SEM. The actual values were 179±19 ng/ml for SPF mice, 105±25 ng/ml for the GF group and 167±50 ng/ml for CBUT mice *P<0.05 as assessed with the Student t-Test for independent data. (c) Levels of serum FSH as determined by ELISA. Each bar represents mean ± SEM. The actual values were 169±18 ng/ml for SPF mice, 95±26 ng/ml for the GF group and 123±9 ng/ml for CBUT mice *P<0.05 as assessed by the Student t-Test for independent data. (B) The relative expression of mRNA species encoding steroidogenic and Leydig cell proteins **normalized to the housekeeping levels of** ***18s rRNA*** **and** ***Arbpa/Rplp0*** **mRNA** in the testes of 8–10 weeks SPF, GF and CBUT mice. Each bar represents the mean ± SEM for 6 mice from 3 different litters. *cyp11a1:* cytochrome P450, family 11, subfamily a, polypeptide 1, *cyp19a1:* cytochrome P450, family 19, subfamily a, polypeptide 1, *Hsd3b1:* hydroxy-delta-5-steroid dehydrogenase, 3 beta- and steroid delta-isomerase 1, *Hsd17b11*: hydroxysteroid (17-beta) dehydrogenase 11 and *Insl3:* insulin-like 3. *Arbpa/Rplp0*: ribosomal protein, large, P0.

The intra-testicular level of testosterone in GF mice was found to be significantly lower than in SPF and CBUT mice (Figure 5A a), while the serum levels of LH and FSH were higher in the SPF mice compared to the GF group (Figure 5A b–c). Assessment of the expression of mRNA encoding proteins related to synthesis of testosterone revealed elevated levels of *Hsd3b1*, *Hsd17b11*, *Cyp11a1* and *Insl3* in CBUT mice in comparison to SPF and GF animals. The expression of *Cyp19a1* was not altered by the gut microbiota or CBUT (Figure 5 B).

## Discussion

This study establishes a novel role for the commensal gut microbiota in the regulation of testicular development and function. Absence of the normal microbiota influences the formation and the integrity of the BTB as well as the intra-testicular levels of testosterone and serum levels of LH and FSH.

Postnatal development of the seminiferous tubules in the murine testis involves proliferation of Sertoli cells up to day 18 [24], along with formation of the lumen. In GF mice, lumen formation was reduced at postnatal day 16 and re-colonization with CBUT restored this formation to that of the control (SPF). Since lumen formation is considered to reflect final differentiation of Sertoli cells [25], the observations indicate that products derived from the gut microbiota can influence the maturation process of these cells and subsequently the formation of the BTB. The increased number of Sertoli cells may also add to the observed changes in the BTB.

The permeability and integrity of the BTB was examined by perfusion experiments *in vivo* and by assessment of the tight- and adherens junctional proteins that form contacts between adjacent Sertoli cells. Evans blue, (bound to albumin) diffused from the vasculature- into the adluminal compartment in the GF mice only, indicative of a permeable BTB in the absence of gut microbiota. The necessity of the gut microbiota for a functional BTB was further emphasized by the experiments with recolonization of the gut (CBUT). The gut microbiota has been demonstrated to be crucial for the establishment of the intestinal barrier [26] and we have previously shown that the gut microbiota can influence the permeability of the blood-brain barrier (Braniste *et al.*, in revised version) [13]. These findings suggest a more general involvement of the gut microbiota in the regulation of the blood tissue barriers, similar to what has been proposed for the blood-brain barrier.

Disruption of the BTB has been associated with impaired sperm formation and consequent reduced fertility [27]. Therefore, the normal spermatogenesis and fertility in our GF mice were unexpected. This suggests that the major contribution of the BTB to spermiogenesis is protection against external/environmental factors (e.g., microbial pathogens), rather than to separate the nutritional/hormonal milieu (e.g., lactate formation [28]), into basal and adluminal compartments.

The process of spermatogenesis and the production of good quality sperm are hormonally regulated by a negative feedback loop of the hypothalamus-pituitary-testis axis. Nutritional, socioeconomic, lifestyle and environmental factors (among others) are involved in the regulation of normal spermatogenesis. A large body of evidence has accumulated over the last years, suggesting that the decrease in male fertility (in terms of sperm count and quality, and other changes in male reproductive health) might be due to an increased exposure to environmental toxicants. Several environmental contaminants can mimic the intracellular actions of estrogens and thus target testicular spermatogenesis, steroidogenesis, and the function of both Sertoli and Leydig cells. The gut microbiota is one such potential source of environmental factors/products that has developed an intimate symbiotic relationship with host's physiology. Manipulation of the gut microbiotia through dietary modification, pre- and probiotics can therefore be beneficial for the host's reproductive health. In the current study, colonizing GF mice with CBUT resulted in an increased sperm production, suggesting that bacterial products, e.g. of fermentation, directly or indirectly, can affect the testis.

The increased permeability of the BTB was associated with altered expression of tight- and adherens junction proteins. The transmembrane claudin proteins are essential for the formation of tight junctions, where they create charge-selective pores. To date, 27 members of this family have been characterized in mammals [29], one of which, claudin 11 (Cldn11), has been reported to participate in the formation of tight junctions in Sertoli cells [3], [4]. Deletion of the *cldn 11* gene disrupted the BTB and resulted in sterility [30]. Moreover, the absence of Cldn11 in Sertoli cells impaired spermatogenesis and lead to detachment of these cells from the basement membrane, to loss of their polarity and to transition to a fibroblastic morphology, albeit with a continued expression of markers for Sertoli cell differentiation [31]. However, the expression and localization of Cldn11 in the testis of GF and SPF mice were similar (Figure S2).

Occludin, another transmembrane protein with two extracellular loops is connected to the actin cytoskeleton via the adaptor proteins of the zonula occludens family (ZO-1, ZO-2 and ZO-3). Disruption of the BTB with cadmium [32] was associated with a significant decline in the steady-state levels of occludin [33]. Although knockout mice lacking occludin did not exhibit an obvious change in their BTB [34], *in vivo* knockdown with lentivirus RNAi disrupted the BTB and induced apoptosis of the germ cells [35]. Loss of ZO-2 expression in the testis was also associated with a compromised BTB [36]. Here, the levels of both occludin and ZO-2 in the GF testis were substantially lower than in SPF mice, with no difference with respect to two other zonula occludens proteins, ZO-1 and ZO-3 (Figure S2). These observations suggest more specific regulation, direct or indirect, of different components of the tight junctions by products generated by the microbiome.

In the present investigation, the gut microbiome did not only influence tight junctions. The levels of a trans-membrane component of the adherens junction complex, E-cadherin, was also reduced in GF mice, whereas the levels of an adaptor protein involved in intracellular signal transduction, β-catenin, was unaffected in testis tissue specimens obtained from GF mice. An age-related reduction of the components of adherens junctions (mRNA and protein) in aging mice was recently described by Paul and Robaire [37]. Furthermore, this demise of the BTB was associated with an increased permeability for a FITC tracer between the ages of 18 to 24 months. Their conclusion was that the disruption of the BTB could expose the germ cells and the seminiferous tubules to hazardous substances and immune cells that can explain the decrease in spermatogenesis observed with increasing age. Their results emphasize the need for a functional and intact BTB in a “hostile” environment in order to protect male fertility.

It was recently shown that testosterone promotes the expression of tight junction proteins at the BTB [38]. For example, knockout experiments of the androgen receptors in Sertoli cells significantly impaired the BTB and also reduced the levels of occludin [39], [40]. An earlier study reported that the absence of gut microbiota influenced testosterone levels [41] in accordance with our observation of a lower intratesticular level of testosterone in GF mice compared to SPF and CBUT mice. A recent study demonstrated that dietary supplementation of the probiotics *Lactobacillus reuteri* increased and restored testosterone levels in aging mice [42]. We could not detect any differences in the expression levels of genes specific for Leydig cell or the levels of mRNA encoding enzymes involved in testosterone production (e.g., *Insl3, Hsd3b1, Hsd17b11, cyp11a1 and cyp19a*). Surprisingly, colonization with CBUT enhanced expression of these genes. One explanation for the normalization of testosterone levels is that bacterial colonization can affect cholesterol and bile acid metabolism in the gut, resulting in an increased reabsorption of substrate for steroid synthesis from the intestine [17]. Furthermore, testosterone levels are also controlled by gonadotropins secreted from the pituitary glands and bacterial metabolites such as butyrate have been shown to increase the levels of LH [43] and FSH [44]. The levels of gonadotropins were also lower in GF mice, but were not significantly normalized by colonization with CBUT, which secrete very high levels of butyrate. This suggests that butyrate most likely regulates testosterone production at the testicular level by stimulation of gene expression in Leydig cells and with little or no effect at the pituitary- hypothalamic levels.

## Conclusions

The present study provides new insights into the unique involvement of the gut microbiota in the regulation of the endocrine functions of the testis and the integrity of the BTB. A delayed development of or a disruption of the BTB will be harmful by allowing the access for immune cells, and other exogenous (environmental) factors to the testis with deleterious effects on germ cells and spermatogenesis. The results also open the possibility that alteration of the gut microbiota in adult life might promote testicular pathologies and decreased fertility.

## Supporting Information

Figure S1 **Immunohistochemistry staining for (A) Vimentin in the testis of 8–10 weeks old SPF, GF and CBUT mice followed by (B) percentage of Sertoli cells.** The values shown are means ± SEM (n = 6) ***P<0.001 calculated by One-way ANOVA.
(TIF)

Figure S2 **Immunofluorescent staining for ZO-1, ZO-3 and claudin-11 (all bright red) in the testis of 8–10 weeks old SPF and GF mice.** Scale bar = 20 µm.
(TIF)

Table S1 **Primer sequences for real time PCR.**
(TIF)

Table S2 **Primary and secondary antibodies.**
(TIF)
