# Supplementary figures and images for: The Gut Microbiota and Developmental Programming of the Testis in Mice
Source: PLoS One. 2014 Aug 13;9(8):e103809. doi: 10.1371/journal.pone.0103809 (PMC4132106; doi:10.1371/journal.pone.0103809)

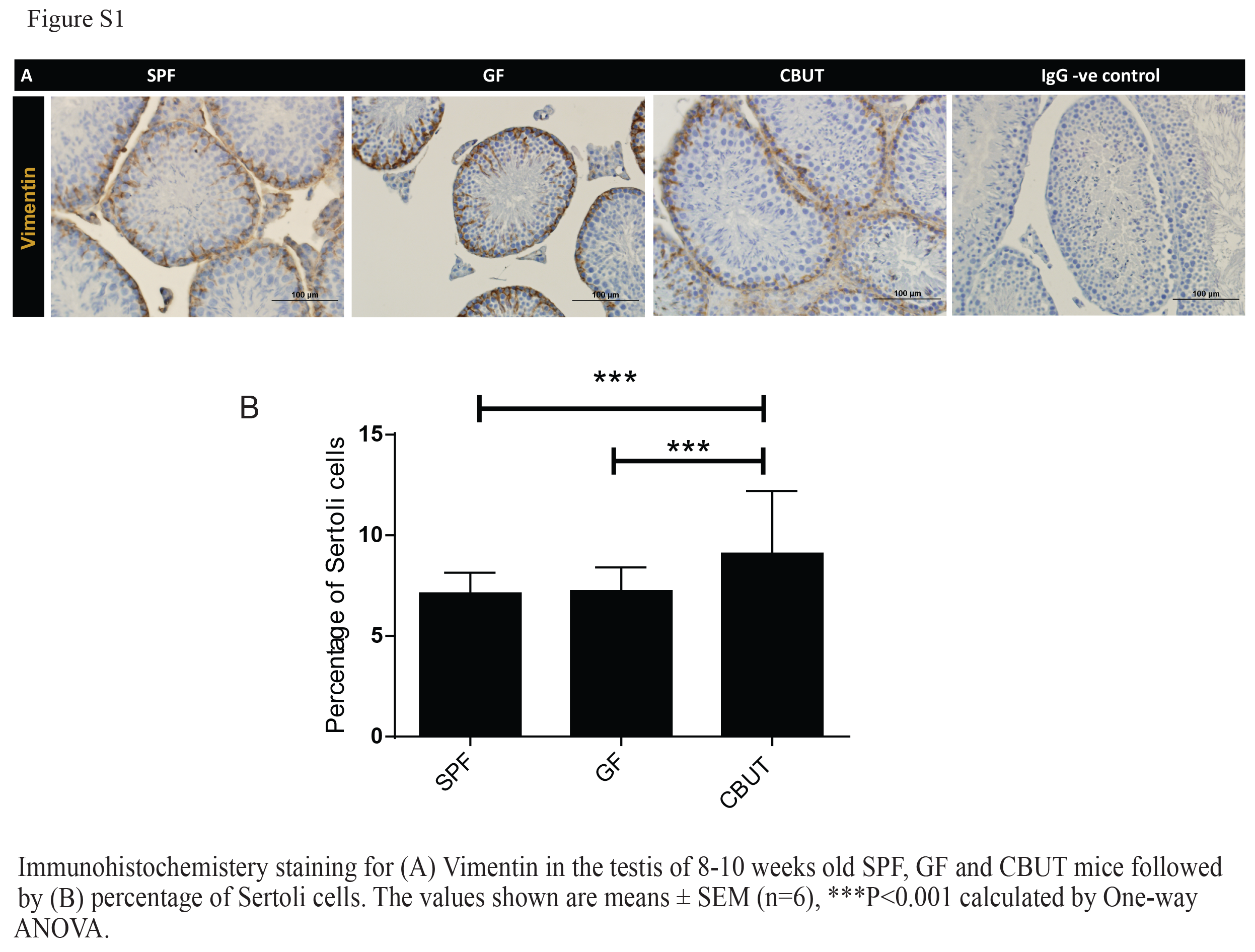

Supplement: Figure S1 — Immunohistochemistry staining for (A) Vimentin in the testis of 8–10 weeks old SPF, GF and CBUT mice followed by (B) percentage of Sertoli cells. The values shown are means ± SEM (n = 6) ***P<0.001 calculated by One-way ANOVA. (TIF) [file pone.0103809.s001.tif]

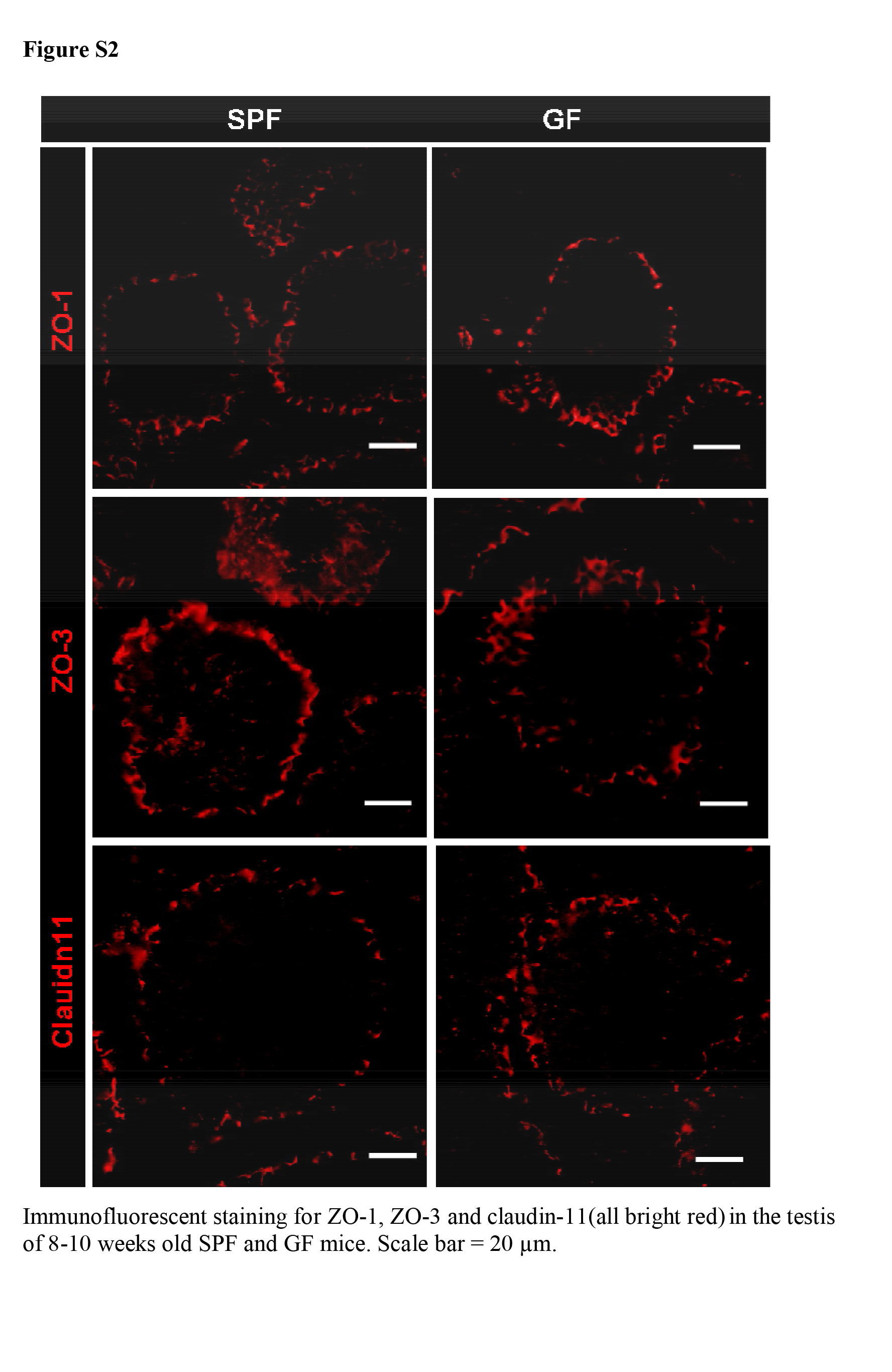

Supplement: Figure S2 — Immunofluorescent staining for ZO-1, ZO-3 and claudin-11 (all bright red) in the testis of 8–10 weeks old SPF and GF mice. Scale bar = 20 µm. (TIF) [file pone.0103809.s002.tif]

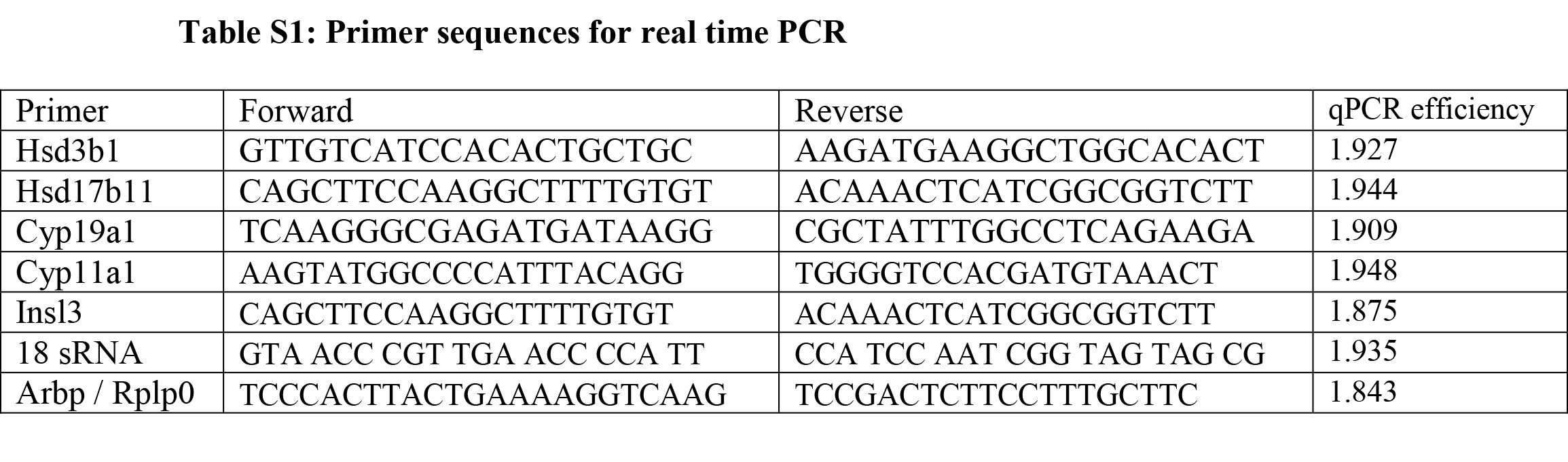

Supplement: Table S1 — Primer sequences for real time PCR. (TIF) [file pone.0103809.s003.tif]

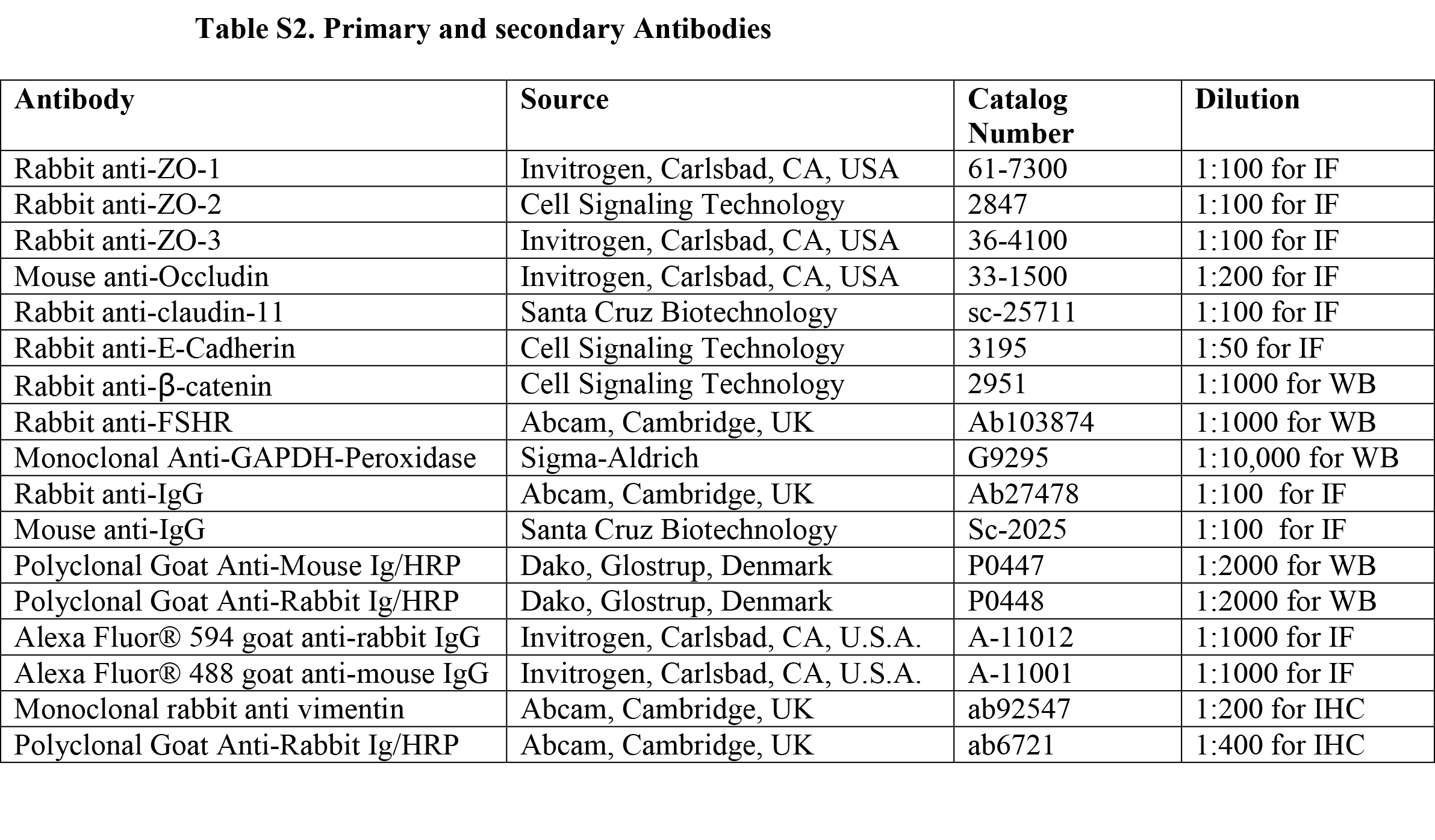

Supplement: Table S2 — Primary and secondary antibodies. (TIF) [file pone.0103809.s004.tif]
